# Survival of Complete Coverage Tooth-Retained Fixed Lithium Disilicate Prostheses: A Systematic Review

**DOI:** 10.3390/medicina59010095

**Published:** 2022-12-31

**Authors:** Abdulmohsen AlMashaan, Abdullah Aldakheel

**Affiliations:** 1 Consultant of Esthetic & Restorative Dentistry, Prince Sultan Military Medical City, Riyadh 11159, Saudi Arabia; 2 Department of Restorative Dentistry, Prince Sultan Military Medical City, Riyadh 11159, Saudi Arabia

**Keywords:** ceramic, lithium disilicate, success, survival, systematic review

## Abstract

*Background and objectives*: Porcelain-fused-to-metal (PFM) prostheses are considered the gold standard for the replacement of missing teeth, however, these have several drawbacks. Therefore, lithium disilicate (LDS) prostheses have been introduced for the construction of fixed crowns and bridges. The aim of this systematic review was to ascertain the long-term survival of LDS fixed prostheses in comparison to other materials. *Materials and methods*: The focused question was ‘In patients who have undergone prosthodontic treatment (participants), what are the overall survival rate of lithium disilicate (LDS) crowns and fixed bridges; and how do they relate to survival rates of non-LDS similar restoration are the survival and com-plication rates (outcomes) of LDS-based fixed prostheses with complete coverage (intervention) higher or lower when compared to non-LDS materials (controls)?’. An electronic search was conducted in PubMED/Medline, EMBASE, Google Scholar, and ClinicalTrials.gov for articles published between January 2006 and August 2022 using appropriate MeSH terms and keywords. The following types of studies were included: (1) All types of prospective clinical studies; (2) Clinical studies focusing on the survival of fixed LDS bridges and crowns; (3) Studies using natural teeth with complete coverage as abutment for fixed LDS bridges and crowns; and (4) Studies in English. The following studies were excluded: (1) Laboratory/in vitro studies and studies on LDS prostheses with no description of outcomes or survival rates; (2) Commentaries; (3) Letters to the editor; (4) Reviews; and (5) Internal data from manufacturers. The data from included studies were extracted and the risk of bias was assessed within the studies using ROBINS-I. *Results*: A total of 25 studies were included in this systematic review. The overall 5-year and 10-year survival rates were of 95–100% and 71.4–100%, respectively. Generally, three-unit bridges had a significantly lower survival rate over 5 and 10 years compared to single-unit crowns. Overall, the risk of bias in the included studies was moderate. *Conclusions*: The LDS-based complete coverage prostheses have a survival rate ranging between 48.6% and 100%. Furthermore, due to the lack of comparative studies, the long-term function and survival of LDS prostheses compared to other material prosthesis (PFM and ZrO) is debatable.

## 1. Introduction

Porcelain-fused-to-metal (PFM) prostheses have conventionally been the treatment of choice for missing teeth, primarily due to their commendable mechanical and biological properties. Although PFM prostheses have shown 10-year survival rates of more than 95% [1], they have several limitations. Firstly, their esthetics may be compromised due to the visibility of the metal framework at gingival margins or the opaque ceramic layer that is required to mask the metal substructure [2]. Therefore, more recently, ceramic-only crowns and bridges have gained popularity. Although all-ceramic prostheses do significantly overcome the esthetic limitations of PFM crowns and bridges, they possess numerous mechanical limitations and inadequacies in their physical strength. Porcelain is traditionally a fragile material and hence fractures easily in thin cross-sections [3]. To overcome these shortcomings, stronger materials such as polycrystalline zirconia and lithium disilicate (LDS) glass ceramics have been introduced as core and surface materials for dental prostheses [4]. Nevertheless, the zirconia substructure can still result in an opaque crown if used underneath porcelain [5].

Lithium disilicate (Li_2_Si_2_O_5_; LDS) is a relatively newer material that has been used in the manufacture of fixed dental prostheses [6]. Being a glass-ceramic, it is machinable, has excellent translucency, and possesses exceptional mechanical properties. Therefore, recent research has focused on using LDS as a material for the manufacture of pressed and CAD/CAM dental prostheses and it has been suggested that LDS prostheses perform better than zirconia [7]. IPS Empress II (Ivoclar Vivadent, Schaan, Liechtenstein), an LDS-based prostheses system that has gained popularity, was introduced as a successor to IPS Empress I (leucite-based ceramic) (6). The LDS-based IPS Empress II had three times better flexural strength than leucite ceramic and was indicated for inlay, onlay, crowns, veneers and anterior three-unit bridges [7,8,9,10,11,12,13]. In the last decade, further development and research has resulted in the formulation of newer LD ceramics (IPS Emax Press & IPS Emax CAD, Ivoclar Vivadent), showing improvements in physical properties and translucency [6,14]. 

For a prosthesis to be successful, it should not only restore esthetics, but it should provide masticatory efficiency without failure. A prostheses may fail due to the chipping of the layering veneer, debonding from the tooth structure or flexural fractures [8]. In a recent study, LDS prostheses were indicated to possess a 10-year survival rate of more than 90% [9,10]. Nevertheless, in another study, the 15-year survival of LDS prostheses was recorded as approximately 49% [10]. As these studies have shown controversial findings in terms of survival rates for LDS ceramic restorations [9,10], it is critical to assess the survival rates of LDS ceramics by summarizing the clinical survival rates data in a systematic review as well as evaluate the factors influencing the survival of these restorations. Therefore, the aims of this systematic review were to summarize the overall survival of fixed LDS dental prostheses and to critically appraise the literature that focuses on their survival rate.

## 2. Materials and Methods

### 2.1. Focused Question

A focused question was constructed in accordance with the Participants, Intervention, Control and Outcomes (PICO) protocol provided in the Preferred Reporting Items for Systemic Reviews and Meta-analysis (PRISMA) guidelines [11]. The focused question was: ‘In patients who have undergone prosthodontic treatment (participants), are the survival and complication rates (outcomes) of LDS-based fixed prostheses with complete coverage (intervention) higher or lower when compared to non-LDS materials (controls)?’

### 2.2. Literature Selection Criteria

Prior to commencing the literature search, the investigators agreed on inclusion and exclusion criteria for the literature. The following types of studies were included: (1) All types of prospective clinical studies; (2) Clinical studies focusing on the survival of fixed LDS bridges and crowns; (3) Studies using natural teeth with complete coverage as abutment for fixed LDS bridges and crowns; and (4) Studies in English. The following studies were excluded: (1) Studies on LDS prostheses with no description of the outcomes or survival rates; (2) Commentaries; (3) Letters to the editor; and (4) Reviews; and (5) Internal data from manufacturers. 

### 2.3. Literature Search

**Figure 1:**
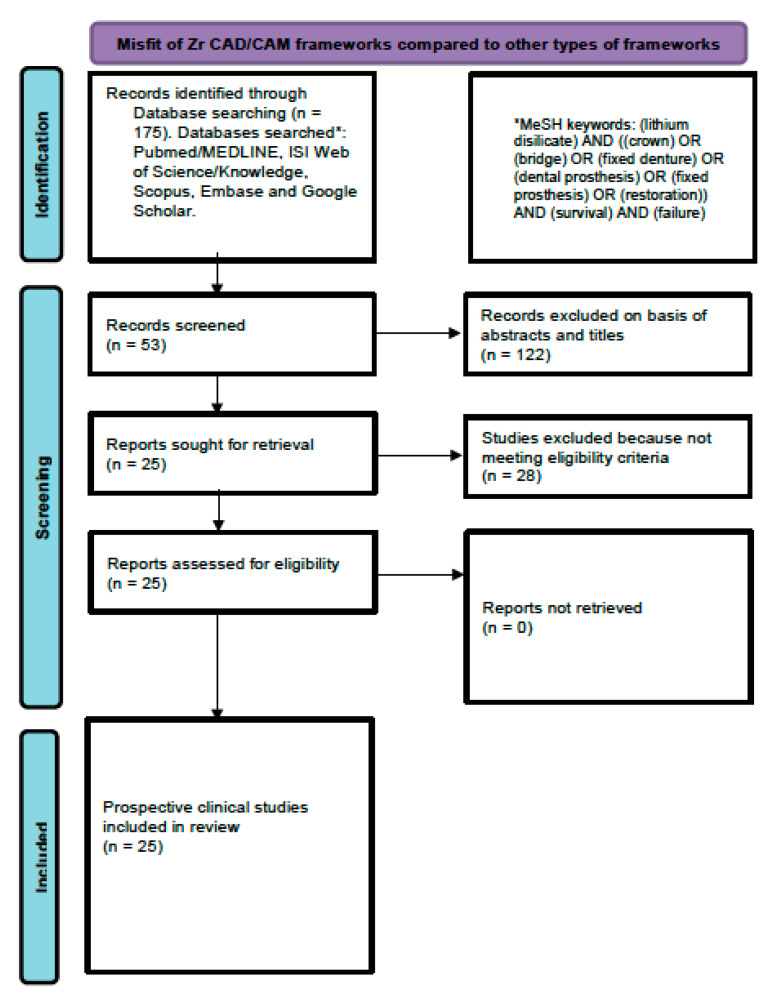
PRISMA flow diagram for the literature search employed for this review.

An electronic search was conducted on the following databases and registers: PubMED/Medline, EMBASE, Google Scholar, and ClinicalTrials.gov for articles published between January 2006 (the year in which fixed LDS prostheses were introduced) and August 2022. The following medical subject heading (MeSH) terms were used: (lithium disilicate) [MeSH] AND ((crown) [MeSH] OR (bridge) [MeSH] OR (fixed denture) [MeSH] OR (dental prosthesis) [MeSH] OR (fixed prosthesis) [MeSH] OR (restoration)) [MeSH] AND (survival) AND (failure). Following the primary literature search, any irrelevant articles were excluded based on titles and abstracts. The full texts of articles, which had the potential to be included in the review, were downloaded. Furthermore, a hand-search of the following journals was performed: *Journal of Prosthodontic Research*, *International of Prosthodontics*, *Journal of Prosthetic Dentistry*, *Dental Materials*, *Journal of Esthetic and Restorative Dentistry*, and *Journal of Prosthodontics*. Additionally, the reference lists of the downloaded full-texts were scanned to find any additional articles meeting our inclusion criteria. The ‘gray literature’ was searched with the assistance of the library services at King Saud University and via the filters and limitations on Google and duplicate studies were eliminated. The search was carried out by two investigators (A.A.M. and A.A.D.) independently and an inter-examiner reliability score (κ) was calculated. Any disagreements were solved by discussion. The literature search strategy is summarized in Figure 1.

### 2.4. Data Extraction

Each investigator extracted data from the included studies corresponding to the following general categories: study design, number of patients, number of prostheses or restorations placed, number of female patients, age range and/or mean of the patients, the prostheses design and number of prostheses used in each experimental and control group (if applicable), and the maximum observation time of the study. The data were categorized and entered into a table (Table 1). Furthermore, the following characteristics specific to the prostheses were entered in another table (Table 2): abutment tooth vitality, experience of the clinicians performing the procedures, fabrication procedure, type of LDS used (monolithic or bilayered), percentage of the repairs needed during the observation period, and the overall survival rate. Any missing data were retrieved by contacting the corresponding authors of the included studies. The data extraction was independently conducted by the aforementioned investigators (A.A.M. and A.A.D.). The data were extracted on to a Microsoft Excel worksheet. Any disagreements were solved by discussion and the extracted data were independently validated by a third subject-matter expert.

### 2.5. Risk of Bias Assessment

The Risk of Bias in Non-Randomized Studies of Intervention (ROBINS-I) developed by Cochrane [12] was used to assess the relative levels of bias in the included studies. Briefly, the following possible sources of bias were assessed to assign each study an overall level of bias: confounding, selection, classification of interventions, deviation of intended interventions, missing data, outcomes, and selective reporting.

## 3. Results

### 3.1. Literature Search

The primary search resulted in 175 studies. After the exclusion of 122 studies based on titles and abstracts, the full texts of 53 studies were downloaded for potential eligibility. Twenty-eight studies were excluded because of the following features: retrospective studies (n = 19), inlays used (n = 3), onlays used (n = 4), partial coverage (n = 1), and systematic review (n = 1). No additional studies were found upon hand-searching or in the grey literature. Therefore, 25 prospective clinical studies were included in this review [9,10,13,14,15,16,17,18,19,20,21,22,23,24,25,26,27,28,29,30,31,32,33,34,35]. Of these 25 studies, six studies were randomized controlled trials (RCTs) [18,20,21,27,33,35] and 19 were non-randomized prospective studies [9,10,13,14,15,16,17,19,22,23,24,25,26,28,29,30,31,32,34]. The inter-examiner reliability score (κ) was calculated as 0.87. No duplicates were found.

### 3.2. General Characteristics of Included Studies

The number of patients ranged from 6 to 2392 [9,10,13,14,15,16,17,18,19,20,21,22,23,24,25,26,27,28,29,30,31,32,33,34,35]. In 15 studies, the number of female patients were reported to range between 2 and 436 [9,10,13,14,15,16,17,18,19,20,21,22,23,24,25,26,27,28,29,30,31,32,33,34,35]. In two studies, female patients were reported as percentages, which were 60.5% [26] and 68% [30], and in seven studies, and the gender of the patients was not reported [15,18,19,21,24,28,33]. The ages of the included patients ranged between 20 and 99 years [9,10,13,14,16,17,20,22,23,24,25,26,27,29,30,31,32,34,35] by 20 studies and four studies did not report the ages [18,19,21,28,33]. The observation time (total follow-up duration) ranged between 1 and 15 years [9,10,13,14,15,16,17,18,19,20,21,22,23,24,25,26,27,28,29,30,31,32,33,34,35]. Seventeen studies included an LDS prosthesis in their study group compared to non-LDS prostheses [10,13,14,17,18,20,21,22,23,24,25,26,27,30,32,34,35], but in eight studies, a comparison group was not included [9,15,16,19,28,29,31,33]. The study groups, along with rest of the general characteristics of the studies, are provided in Table 1.

**Table 1:** General characteristics of the included studies.

Authors, Year	Study Design	Patients (n)	Prostheses/Restoration (n)	Female (n; %)	Age Range; Mean Age (Years)	Prosthesis Design and Group(s) (n)	Maximum Observation Time
Wolfart et al., 2005 [13]	Prospective	68	81	n= 38	20–68	1. LDS 3-unit FPD (n = 36) 2. LDS 3-unit FPD (inlay retained; n = 45)	5 years
Marquardt and Strub, 2006 [14]	Prospective	43	58	n = 19	22–65	1. LDS SC (n = 27) 2. LDS 3-unit FDP (n = 31)	5 years
Toksavul and Toman et al., 2007 [28]	Prospective	21	79	NR	NR	LDS SC (n = 79)	5 years
Esquivel-Upshaw et al., 2008 [16]	Prospective	21	30	n = 18	30–62	LDS 3-unit FPD (n = 30)	2 and 4 years
Wolfart et al., 2009 [19]	Prospective	28	33	17	47.9; 32–64	LDS 3-unit FPD (n = 33)	8 years
Fasbinder et al., 2010 [19]	Prospective	43	62	NA	NA	LDS SC (n = 62)	2 years
Etman and Woolford 2010 [18]	RCT	48	90	NA	NA	1. LDS (n = 30) 2. PFM (n = 30) 3. Alumina-coping (n = 30)	3 years
Makarouna et al., 2011 [20]	RCT	37	37	n = 23	47	1. 3-unit LDS FPD (n = 18) 2. PFM (n = 19)	6 years
Cortellini and Canale 2012 [9]	Prospective	76	235	n = 44	36; 20–61	LDS (n = 235)	3 years
Kern et al., 2012 [22]	Prospective	28	36	n = 17	47.5	SC LDS 1. GIC cement (n = 19) 2. RC cement (n = 17)	10 years
Esquivel-Upshaw et al., 2012 [21]	RCT	31	36	NR	NR	SC 1. PFM (n = 12) 2. LDS glaze only (n = 12) 3. LDS + glass ceramic veneer (n = 12)	3 years
Reich and Schierz 2012 [23]	Prospective	34	41	N = 21	46.5; 26.2–73.8	LDS SC (n = 41)	51 months
Gerht et al., 2013 [25]	Prospective	41	104	n = 26	34	Anterior LDS SC (n = 104)	9 years
Solá-Ruiz et al., 2013 [24]	Prospective	19	21	NR	NR	3-unit FPD (n = 21)	10 years
Rauch et al., 2013 [26]	Prospective	34	41	68%	52.2; 32.9–79.9	Posterior SC (n = 41)	6 years
Toksavul and Toman et al., 2015 [28]	Prospective	34	121	NR	NR	LDS SC (n = 121)	9 years
Grohmann et al., 2015 [27]	RCT	60	60	n = 33	52	Posterior 3-unit FPD 1. LDS (n = 30) 2. ZrO2 (n = 30)	1 year
Aziz et al., 2019 [7]	Prospective	32	40	n = 20	29–79; 50.4	LDS SC (n = 40)	4 years
Malament et al., 2019 [30]	Prospective	556	1960	60.5%	17–97	LDS (n = 1960): SC; 3-unit FPD; cantilevered anterior; foundation restorations	10 years
Garling et al., 2019 [10]	Prospective	28	36	17	47.5	3-unit LDS (n = 36): Anterior 3-unit FPD (n = 6); Posterior 3-unit FPD (n = 30)	15 years
Liebermann et al., 2020 [32]	Prospective	6	40	2	42.2 ± 4.7	Anterior full coverage SC LDS (n = 40)	8 years
Aziz et al., 2020 [31]	Prospective	189	210	n = 121	56.3 ±13.83 (28–88)	LDS SC	6 years
Gardell et al., 2021 [33]	RCT	44	60	NR	NR	1. LDS SC (n = 30)	3 years
Malament et al., 2021 [34]	Prospective	2392	2392	n = 436	20–99	1. Complete coverage LDS SC (n = 1782) 2. Partial coverage LDS SC (n = 610)	16.9 years
Hammoudi et al., 2022 [35]	RCT	62 (with tooth wear)	713	n = 17	44.8; 25–63	1. LDS SC (n = 362) 2. ZrO2 (351)	6 years

RCT, randomized controlled trial; LDS, lithium disilicate; ZrO_2_; SC, single crown; FPD, fixed partial denture; PFM, porcelain fused to metal.

### 3.3. Prosthodontic Variables and Outcomes of the Included Studies

In six studies, only vital abutments were used [10,13,16,18,22,33] and in eight studies, both vital and non-vital (endodontically treated) abutments were used [15,24,25,26,27,29,31,35]. In 11 studies, the vitality of the abutments was not reported [9,14,15,17,19,20,21,23,28,30,32]. In 19 studies, the resin composite cements were used to retain the prostheses [9,13,14,15,18,19,20,21,23,24,26,27,28,29,30,31,32,33,34,35], while in five studies, both the resin composites and glass ionomer cements were used [10,16,17,22,25]. The experience of the clinicians performing the prosthodontic treatment was described in only five studies [13,17,25,29,31] and among these, the clinicians were general practitioners experienced between 1 and 5 years in two studies [13,17], trained by the LDS manufacturer in one study [25], and were final-year dental students in two studies [29,31]. The prostheses were constructed via the lost-wax technique in 18 studies [10,13,14,15,16,17,18,20,21,22,24,25,28,30,32,33,34,35], CAD/CAM was used in six studies [19,23,26,27,29,31], and both were in used one study [9]. In 22 studies, monolithic LDS was used in the construction of the prostheses [9,10,13,14,15,16,17,18,19,22,23,24,25,26,27,28,29,31,32,33,34,35], whilst in two studies, both monolithic and bilayered LDS were used [21,30], and in one study, the type of LDS was not recorded [20]. The complication rates ranged from less than 1% to 46% and the survival rates ranged between 62.7% and 100% [9,10,13,14,15,16,17,18,19,20,21,22,23,24,25,26,27,28,29,30,31,32,33,34,35]. The detailed prosthodontic variables and outcomes are presented in Table 2.

**Table 2:** Other characteristics, survival rates, and outcomes of the included studies.

Authors et al., Year	Abutment Vitality	Cement/Adhesive	Clinician Type/Experience	Fabrication	Type of LDS	Repair Rate (%; Years)	Survival Rate (%; Years) Outcomes
Wolfart et al., 2005 [13]	Vital	GIC	GP; 1–5 years	Lost-wax	Monolithic	2.7%/4 years	100%/4 years
Marquardt and Strub, 2006 [14]	NR	RC	NR	Lost-wax	Monolithic	NR	Crowns: 100%/5 years FPD: 70%/5 years
Toksavul and Toman et al., 2007 [28]	NR	RC	NR	Lost-wax	Monolithic	<1%/5 years	95.21%/5 years
Esquivel-Upshaw et al., 2008 [16]	Vital	Group 1: RMGIC Group 2: RC	NR	Lost-wax	Monolithic	NR	RMGIC: 72.7%/4 years RC: 76.9%/4 years NS
Wolfart et al., 2009 [19]	NR	Group 1: GIC Group 2: RC	GP; mean experience: 3 years	Lost-wax	Monolithic	Overall: 13%/8 years GIC: 20%/8 years RC: 6%/8 years NS	Overall: 93%/8 years GIC: 100%/8 years RC: 85%/8 years NS
Fasbinder et al., 2010 [19]	NR	RC	NR	CAD/CAM	Monolithic	None/2 years	100%/2 years
Etman and Woolford 2010 [18]	Vital	RC	NR	Lost-wax	Monolithic	All-ceramic: 6.6%/3 years LDS: none/3 years PFM: 3.3%/3 years	All groups: 100%/3 years
Makarouna et al., 2011 [20]	NR	GIC	NR	Lost-wax	NR	LDS: 46%/6 years PFM: 11%/6 years	LDS: 62.7 ± 12.1%/6 years PFM: 94.7 ± 5.1%/6 years
Cortellini and Canale 2012 [9]	NR	RC	NR	Lost-wax; CAD/CAM	Monolithic	Pressed: n = <1%/6 years CAD/CAM: none/6 years	Both groups: 100%/6 years
Kern et al., 2012 [22]	Vital	GIC RC	NR	Lost-wax	Monolithic	GIC: n = 5 RC: n = 10	Overall: 90.8%/10 years
Esquivel-Upshaw et al., 2012 [21]	NR	RC	NR	Lost-wax	Monolithic and bilayered	None	Overall: 100%/3 years NS difference between monolithic and bilayered
Reich and Schierz 2012 [23]	NR	RC	NR	CAD/CAM	Monolithic	Overall: 16.6%/4 years	Overall: 96.3%/4 years
Gerht et al., 2013 [25]	Vital/non-vital	GIC and RC	Trained by manufacturer	Lost-wax	Monolithic	Overall: 5.4%/9 years	Overall: 94.8%/9 years
Solá-Ruiz et al., 2013 [24]	Vital/non-vital	RC	NR	Lost-wax	Monolithic	NR	Overall: 71.4%/10 years
Rauch et al., 2013 [26]	Vital (n = 24)/non-vital (n = 17)	RC	NR	CAD/CAM	Monolithic	Overall: 14.6%/6 years	87.6%/years
Toksavul and Toman et al., 2015 [28]	Vital, n = 110 Non-vital, n = 11	RC	NR	Lost-wax	Monolithic	NR	87.1%/9 years
Grohmann et al., 2015 [27]	Vital/non-vital	RC	NR	CAD/CAM	Monolithic	ZrO2: 10%/1 year LDS: 10%/1 year	100 %/1 year NS difference between both groups.
Aziz et al., 2019 [7]	Vital (n = 33) Non-vital (n = 7)	RC	Final-year dental students	CAD/CAM	Monolithic	LDS: none/4 years	95%/4 years
Malament et al., 2019 [30]	NR	RC	NR	Lost-wax	Monolithic (n = 1410) and bi-layered (n = 550)	<1 %/10 years	Monolithic: 96.5/10.4 years Bilayered: 100%/7.9 years p < 0.05
Garling et al., 2019 [10]	Vital	GIC (n = 19) RC (n = 17)	NR	Lost-wax	Monolithic	NR	48.6%/15 years
Liebermann et al., 2020 [32]	NR	RC	NR	Lost-wax	Monolithic	12.5%/11 years	100 %/11 years
Aziz et al., 2020 [31]	Vital/non-vital	RC	Final-year dental students	CAD/CAM	Monolithic	13.3%/6 years	93 %/6 years
Gardell et al., 2021 [33]	Vital	RC	GP; experience not stated	Lost-wax	Monolithic	ZrO2: 20%/3 years LDS: 11.3%/3 years NS	ZrO2: 93.3%/3 years LDS: 100%/3 years NS
Malament et al., 2021 [34]	NR	RC	NR	Lost-wax	Monolithic	<1%/16.9 years (complete and partial coverage)	96.49%/16.9 years
Hammoudi et al., 2022 [35]	Vital (n = 675) Non-vital (n = 38)	RC	NR	Lost-wax	Monolithic	ZrO2: <1%/6 years LDS: 1.4%/6 years	99.7/6 years (ZrO2 and LDS)

GIC, glass ionomer cement; NA, not applicable; NR, not recorded; RC, resin composite cement; RMGIC, resin-modified glass ionomer cement; ZrO_2_, zirconia.

### 3.4. Factors Affecting the Survival of LD Restorations

Overall, it was observed that LDS prostheses survived better on vital teeth than on devitalized teeth [13,17]. The type of material or coverage did not have a significant impact on the survival of the prostheses [34]. Similarly, the position of the prostheses had no significant impact on the survival or complications of the prostheses [9]. It was also observed that LDS-based FDPs had a failure rate of 52.4% after 15 years [10], indicating that LDS prostheses with a longer span have a lower survival rate compared to single crowns. The majority of the fractures of the FDPs occurred at the connectors [24]. In another study, complete-coverage LDS restorations had a significantly higher survival rate compared to inlays [15].

### 3.5. Results of the Quality Assessment

In 15 studies, bias due to confounding variables was estimated as ‘high’ [9,13,14,15,16,17,18,20,21,25,29,31,33,34]. In 20 studies, the selection bias was estimated as ‘high’ [9,10,13,14,15,16,17,18,19,20,22,24,25,28,29,31,32,34,35] and in 5 studies, the selection bias was estimated as ‘moderate’ [21,23,25,30,33]. In all the studies, bias resulting from the misclassification of interventions or from deviation from intended interventions was estimated to be as ‘low’ [9,10,13,14,15,16,17,18,19,20,21,22,23,24,25,26,27,28,29,30,31,32,33,34,35]. Bias resulting from missing data was estimated to be ‘high’ in 10 studies [9,13,14,15,16,17,19,20,22,29,32], ‘moderate’ in 6 studies [16,23,24,26,29], and ‘low’ in 9 studies [10,25,27,28,30,33,34,35]. Bias within the outcomes was graded as ‘high’ in 14 studies [9,10,13,14,17,19,20,21,22,24,26,30,31,32], ‘moderate’ in 4 studies [16,23,28,29], and ‘low’ in 7 studies [15,18,25,27,33,34,35]. Bias due to selective reporting was graded ‘high’ in 5 studies [9,14,20,21,32], ‘moderate’ in 7 studies [10,13,16,22,23,29,31], and ‘low’ in 13 studies [15,17,18,19,24,25,26,27,28,30,33,34,35]. The risk of bias assessment resulted in 5 studies receiving a total bias level of ‘High’ [9,13,14,20,31], 12 studies having a ‘Moderate’ level of bias [10,15,16,17,19,21,22,23,26,29,30,32], and 8 studies receiving an overall bias level of ‘Low’ [18,24,25,27,28,33,34,35]. The individual criteria and their respective grades, along with the overall qualities of studies, are listed in Table 3.

## 4. Discussion

The results from the present study indicate that LDS complete coverage prostheses have a 5-year and 10-year survival rate of 95–100% [14,15] and 71.4–100% [22,24,30,32], respectively. Nevertheless, results by Marquardt and Strub (2006) have revealed a significantly lower 5-year survival rate for LDS-based fixed partial denture with complete coverage [14], which suggests that LDS is currently more suited to construct single crowns than prostheses with a longer span. This is most likely due to the higher flexural forces experienced by bridges relative to crowns [36]. However, this hypothesis should be considered with caution because there is a lack of comparative studies assessing the comparative survival rates of LDS crowns and bridges. Given this, one study by Hammoudi et al. revealed that the 6-year survival rate of LDS prostheses is as high as 99.7%, which was similar to Zirconia prostheses, even in patients with significant tooth-wear, which is indicative of bruxism [35].

The studies by Wolfart et al. suggested that the survival rate of LDS-based prostheses ranged between 100% after 3 years and 94% after 8 years [13,18,19]. Interestingly, in one study, they also observed that LDS failed at a significantly higher rate when resin composites were used instead of glass ionomer cements as adhesives [19]. Kern et al. [22] demonstrated a higher complication rate in resin composite-retained prostheses in comparison to glass ionomers. Similarly, in the study by Esquivel-Upshaw et al. (2008) [16], approximately 5% of LDS prostheses failed within 5 years of an observation period when resin-modified glass ionomer cements (RMGICs) and resin composites were used. Although these results seem to suggest that conventional glass ionomers are more suitable for use with LDS fixed prostheses, none of the studies included all three major classes of adhesives (conventional glass ionomers, resin-modified glass ionomers, and resin composites) to ascertain a more definitive recommendation regarding the most appropriate adhesive system to retain the prostheses. Indeed, other studies recorded 6- to 10-year survival rates of more than 90% when the resin composites were used [30,31,35] which are in contradiction with previous studies. These results warrant future comparative studies that look at the different adhesive systems to synthesize appropriate guidelines for the retention of LDS prostheses.

Since chronic bruxism has been observed to have detrimental effects on the survival rate of dental prostheses [37], these results suggest that LDS-based prostheses are a promising material for patients with parafunctional oral habits. In the included studies, it was observed that none of the variables had a significant or conclusive effect on the overall survival rate of the prostheses [9,10,13,14,15,16,17,18,19,20,21,22,23,24,25,26,27,28,29,30,31,32,33,34,35]. The type of fabrication (CAD/CAM or lost-wax/laboratory) resulted in comparable survival rates [9,10,13,14,15,16,17,18,19,20,21,22,23,24,25,26,27,28,29,30,31,32,33,34,35]. Nevertheless, the reduced processing times and appointments involved in the chairside CAD/CAM fabrication of the prostheses do indeed present worthwhile advantages when compared to laboratory process dental prostheses [38]. Therefore, we hypothesized that CAD/CAM LDS-based protheses will gain popularity in the future. Moreover, LDS prostheses have been shown to have fracture/complications and survival rates similar to those of Zirconia [27,33,35]. However, to date, only three studies have compared the survival and complication rates of zirconia and LDS [27,33,35], which warrants more research to compare both the materials. Interestingly, only two studies compared the survival or complications of LDS prostheses to those of PFM crowns, considered the ‘gold standard’ [18,20]. In both studies, none of the LDS prostheses experienced any complications or failures, compared to 3.3% PFM prostheses experiencing complications and no failures after 3 years [18]. In the other study, however, LDS prostheses experienced a significantly higher complication rate (46%) and a lower survival rate (62.7%) compared to PFM prostheses (complication rate: 11%; and survival rate 94.7%) after 6 years [20]. The only study observing LDS-based three-unit restorations for 15 years had recorded a survival rate of 48.6% [10], which is lower than the 66.5% of PFM three-unit FDPs reported by previous studies [39]. This suggests that LDS prostheses may survive a lower rate than PFM crowns and bridges, but more studies are needed to ascertain the comparative long-term survival of both types of prostheses. 

Due to the multifactorial failure and complications of dental prostheses, it is difficult to standardize the clinical studies conducted to compare or observe the performances of dental prostheses. However, it has been generally agreed that survival rates lower than 90% are considered poor [8]. Several factors impact the success (or failure) of fixed all-ceramic and PFM prostheses. According to Chadwick et al. [40], these factors are: the type of restoration, size and site of restorations, age, gender, socioeconomic characteristics of the patients, and the oral hygiene status. Furthermore, the age, salary, and experience of the operator also play a role in the overall outcomes of prosthodontic rehabilitation. The majority of studies included in this review did not investigate the effects of these variables on the success or complications of LDS prostheses. Only a handful of studies stated the overall duration or type of clinical experience of the practitioners involved in the clinical phases of prosthodontic rehabilitation [13,17,25,29,31], so future research could focus on the impact of operator experience on the survival of LDS prostheses. Indeed, in two studies, a 4-year survival rate of 93–95% was observed in crown preparations completed by final year students, which indicates that operator experience may not have a significant impact on the outcomes of LDS-based prostheses [29,31]. Studies indicate that the choice of adhesive has no significant overall impact on the functionality or survival of LDS prostheses [16,17,22], mirroring the outcomes observed by a previous systematic review on the survival rate of CAD/CAM-only prostheses [7]. No clear or significant differences were observed due to the tooth position or type in the studies that included these variables, which suggests that LDS-based prostheses can be used for the restoration of any type or position of tooth [9,10,13,14,15,16,17,18,19,20,21,22,23,24,25,26,27,28,29,30,31,32,33,34,35]. In the studies we reviewed, no clear trend was observed in the type of complications—there was equal predisposition of biological (e.g., caries, pain, gingivitis, etc.) and mechanical (fracture, porcelain fracture, etc.) complications [9,10,13,14,15,16,17,18,19,20,21,22,23,24,25,26,27,28,29,30,31,32,33,34,35]. However, in the majority of studies, these complications were apparent 2 years after the cementation of the crown [9,10,13,14,15,16,17,18,19,20,21,22,23,24,25,26,27,28,29,30,31,32,33,34,35].

A strength of this review was that the evaluators were able to include 25 clinical studies which, to the best of our knowledge, is the first time this has been achieved. Nevertheless, due to the heterogeneity in the methodologies and study groups among the included studies, no meta-analysis was conducted in this systematic review, which can be considered a limitation of this study. Therefore, it was not possible to pool the overall outcomes and survival rates of the prostheses. Another limitation was the inability to review non-English papers because the investigators were not proficient in languages other than English or Arabic. Since none of the studies included explicit descriptions of randomization process or blinding, it is difficult to deem them internally valid. The quality assessment of the studies revealed several sources of bias which may have influenced their outcomes. Another major limitation of the included studies was that the majority of them did not look at survival rates exceeding 5 years. Only two studies recorded the complication or survival of LDS prostheses for 10 or more years. Future studies should follow up patients for longer periods of time to determine a more meaningful conclusion regarding the survival of the prostheses. Additionally, the majority of studies did not attempt to reduce the influence of other confounding variables such as parafunctional habits, smoking, or other forms of substance abuse, variables which future studies should include to evaluate their effects on LDS prostheses in comparison to other materials. None of the included studies included a cost-effectiveness evaluation of LDS prostheses so it is unknown whether the associated costs and outcomes involved in their usage is similar to or better than currently available materials. Another worthwhile avenue to look at would be the comparison between the survival rates of LDS prostheses processed by more seasoned clinicians or specialists with those made by general practitioners, fresh dental graduates, or dental students. To date, not many studies have compared the survival or complication rates of LDS with those of other materials such as porcelain, PFM, base metals, and titanium alloys. Therefore, future studies should include these comparison groups. The lack of standardization, suitable comparison groups and inadequate follow-up make the external validity of these studies debatable. Based on these limitations, the characteristics, quality, and outcomes of the included studies, it may be suggested that there is a low level of evidence that the survival and complication rates of LDS-prostheses are similar to those of other materials; further research is strongly advocated before they can be used more widely.

## 5. Conclusions

LDS-based complete coverage prostheses have survival rates ranging between 48% and 100%. Furthermore, due to the lack of comparative studies with sufficient follow-up, the long-term function and survival of LDS prostheses compared to other material prostheses (PFM and ZrO) is debatable.

## Figures and Tables

**Table 3:** Quality assessment results of the included studies (Scale: ROBINS—Cochrane).

	Sources and Levels of Bias							
Study (Author, Year)	Confounding	Selection	Classification of Interventions	Deviation from Intended Intervention	Missing Data	Outcomes	Selective Reporting	Overall Bias
Wolfart et al., 2005	High	High	Low	Low	High	High	Moderate	High
Marquardt and Strub, 2006	High	High	Low	Low	High	High	High	High
Toksavul and Toman et al., 2007	High	High	Low	Low	High	Low	Low	Moderate
Esquivel-Upshaw et al., 2004, 2008	High	High	Low	Low	High	Moderate	Moderate	Moderate
Wolfart et al., 2009	High	High	Low	Low	High	High	Low	Moderate
Fasbinder et al., 2010	Moderate	High	Low	Low	High	High	Low	Moderate
Etman and Woolford 2010	High	High	Low	Low	Moderate	Low	Low	Low
Makarouna et al., 2011	High	High	Low	Low	High	High	High	High
Cortellini and Canale 2012	High	High	Low	Low	High	High	High	High
Kern et al., 2012	Moderate	High	Low	Low	High	High	Moderate	Moderate
Esquivel-Upshaw et al., 2012	High	Moderate	Low	Low	Moderate	High	High	Moderate
Reich and Schierz 2012	Moderate	Moderate	Low	Low	Moderate	Moderate	Moderate	Moderate
Gerht et al., 2013	High	Moderate	Low	Low	Low	Low	Low	Low
Solá-Ruiz et al., 2013	Moderate	High	Low	Low	Moderate	High	Low	Low
Rauch et al., 2013	Moderate	High	Low	Low	Moderate	High	Low	Moderate
Toksavul and Toman et al., 2015	Low	High	Low	Low	Low	Moderate	Low	Low
Grohmann et al., 2015	Low	Low	Low	Low	Low	Low	Low	Low
Aziz et al., 2019	High	High	Low	Low	Moderate	Moderate	Moderate	Moderate
Malament et al., 2019	Moderate	Moderate	Low	Low	Low	High	Low	Moderate
Garling et al., 2019	Moderate	High	Low	Low	Low	High	Moderate	Moderate
Liebermann et al., 2020	Moderate	High	Low	Low	High	High	High	Moderate
Aziz et al., 2020	High	High	Low	Low	High	High	Moderate	High
Gardell et al., 2021	High	Moderate	Low	Low	Low	Low	Low	Low
Malament et al., 2021	High	High	Low	Low	Low	Low	Low	Low
Hammoudi et al., 2022	High	High	Low	Low	Low	Low	Low	Low

## Data Availability

The data are available from the corresponding author.
